# Successful treatment of a case of super-high malignant esophago-tracheal fistula with Sigma fully covered esophageal stent placement: case report

**DOI:** 10.3389/fmed.2025.1533538

**Published:** 2025-03-28

**Authors:** Shupei Li, Qi Zhai, Ya Yang, Huafei Li, Juan Xu, Ying Zhang, Ji Xuan

**Affiliations:** ^1^Department of Gastroenterology, Jinling Clinical Medical College, Nanjing University of Chinese Medicine, Nanjing, China; ^2^Department of Gastroenterology and Hepatology, Jinling Hospital, Affiliated Hospital of Medical School, Nanjing University, Nanjing, China; ^3^Department of Pathology, Jinling Hospital, Affiliated Hospital of Medical School, Nanjing University, Nanjing, China

**Keywords:** esophageal stent placement, esophageal-tracheal fistula, anastomotic stenosis, fully covered metallic stent, case report

## Abstract

**Background:**

Esophageal cancer, as one of the most frequently occurring malignancies globally, often leads to esophageal stenosis and fistulas post-surgery or radiotherapy, significantly impacting patients’ quality of life and prognosis. In particular, super-high esophageal fistulas represent a clinically challenging issue that urgently needs to be addressed.

**Case summary:**

This study reports on a 72-year-old female patient who developed anastomotic stenosis following esophageal cancer surgery. Endoscopic exploration revealed that the esophageal inlet was approximately 17 cm from the incisors, with the anastomotic site located 20 cm from the incisors and being stenotic, rendering endoscope passage impossible. An ultra-thin gastroscope was then used, showing the stenosis at 20–22 cm. Our center successfully managed the high anastomotic stenosis with a Sigma fully covered esophageal stent. Subsequently, the patient experienced anastomotic recurrence and multiple esophageal-tracheal fistulas, which were successfully sealed with the same type of stent. A stent replacement was performed one year later, successfully improving the patient’s quality of life.

**Conclusion:**

This study demonstrates the successful management of a super-high esophageal-tracheal fistula using a Sigma fully covered metallic stent.

## 1 Introduction

Esophageal cancer is the eighth most common cancer and the sixth leading cause of cancer-related death worldwide (1). Currently, surgical resection is the primary treatment option for esophageal cancer patients, often followed by adjuvant radiotherapy and chemotherapy. However, not only radiotherapy but also surgery can lead to esophageal stenosis and fistulas (2). Studies have shown that approximately 6–22% of cases develop esophageal fistulas post-treatment intervention for esophageal cancer, with a very poor prognosis (3). Fully covered esophageal metallic stent placement is currently the safest and most effective method widely used clinically for the treatment of esophageal stenosis and esophageal-tracheal fistulas, providing an effective solution for feeding issues and preventing lung infections (4).

Super-high esophageal fistulas occur in the upper segment of the esophagus, endoscopically located 15–23 cm from the incisors. Due to its special anatomical structure, the placement of metallic stents requires higher technical expertise, and improper handling can lead to numerous complications, such as stent migration, foreign body sensation in the throat, tissue hyperplasia, and inability to remove the stent. The Sigma fully covered esophageal stent has numerous advantages compared to traditional stents. In terms of preventing displacement, its flared upper end, anti-slip barbs, and suspension wire design effectively reduce the risk of displacement. This is crucial in the treatment of super-high esophageal fistula stenosis, ensuring the stable function of the stent. Regarding the reduction of foreign body sensation, the stent features a full silicone membrane coverage and a segmented design. This reduces irritation to the esophageal mucosa, decreases the diameter of the metal wires and the supporting force, significantly enhancing the patient’s comfort and improving the patient’s acceptance of the treatment. When it comes to minimizing tissue hyperplasia, the silicone membrane design of the Sigma stent prevents tissue adhesion and scar formation. It can be smoothly removed even after long-term placement, solving the problems of difficult removal and restenosis caused by tissue hyperplasia in traditional stents. In terms of precise placement, with the aid of an external positioning ruler, this stent can achieve millimeter-level precise positioning. This improves the success rate and safety of the surgery, especially for the placement of super-high position stents.

Currently, in the field of esophageal disease treatment, esophageal stent placement has become an important intervention (4). However, research on the Sigma fully covered esophageal stent is relatively limited. In the English-language literature, only one relevant case report has been published. This report applied a Y-shaped Sigma stent loaded with I^125^ seeds to patients with inoperable malignant main airway obstruction and reported the effectiveness of this stent in tracheal treatment. After the operation, the patients’ airway lumen was restored well, and their Karnofsky Performance Status (KPS) scores and partial pressure of oxygen in arterial blood (PaO₂) were significantly improved (5). Nevertheless, to date, no English-language literature has reported on the application of Sigma stents in the treatment of esophageal diseases. This study aims to fill this gap by describing a case of a patient with a super-high malignant esophago-tracheal fistula who was successfully treated with a Sigma fully covered metallic stent.

## 2 Case presentation

### 2.1 Chief complaints

**Figure 1:**
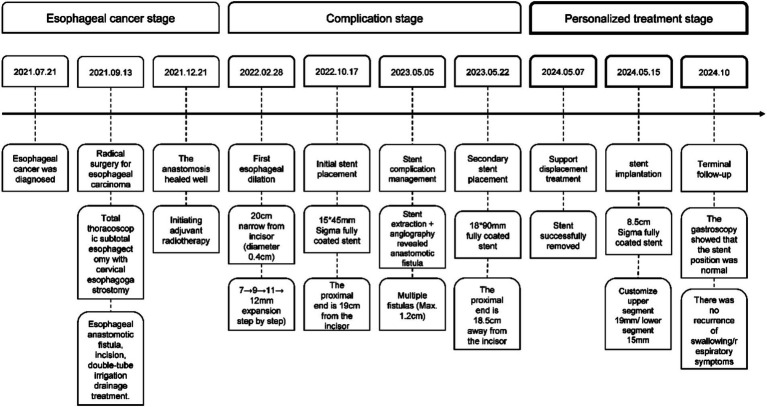
Patient treatment process timeline.

Five days ago, the patient developed eating obstruction, vomiting after drinking water, and coughing (Figure 1).

### 2.2 History of present illness

On May 1, 2024, the patient was admitted due to dysphagia, vomiting even with water intake, and coughing.

**Figure 2:**
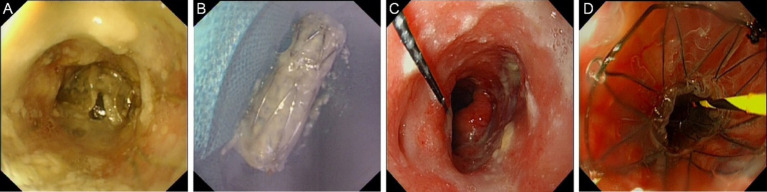
Endoscopic images in May 2023. **(A)** Stent *in-situ* image. **(B)** Stent removal image. **(C)** Fistula image. **(D)** Stent replacement image.

On May 5, 2023, the patient presented with self-reported throat pain and irritative cough and underwent esophageal stent removal (Figures 2A,B). Upper gastrointestinal radiography revealed an anastomotic fistula. After oral administration of meglumine diatrizoate, the anastomotic site mucosa appeared slightly disordered without significant stenosis, and a small amount of contrast agent was seen extravasating into the trachea and thoracic stomach, suggesting an esophageal-tracheal fistula. Endoscopic exploration showed the anastomotic site at approximately 20 cm from the incisors, with fistulas at approximately 21 cm and 22–24 cm from the incisors (Figure 2C), and stenosis had resolved. Pathology at the “esophagus at 22 cm from the incisors” showed inflammatory necrotic granulation tissue and tumor, suggesting possible local recurrence.

**Figure 3:**
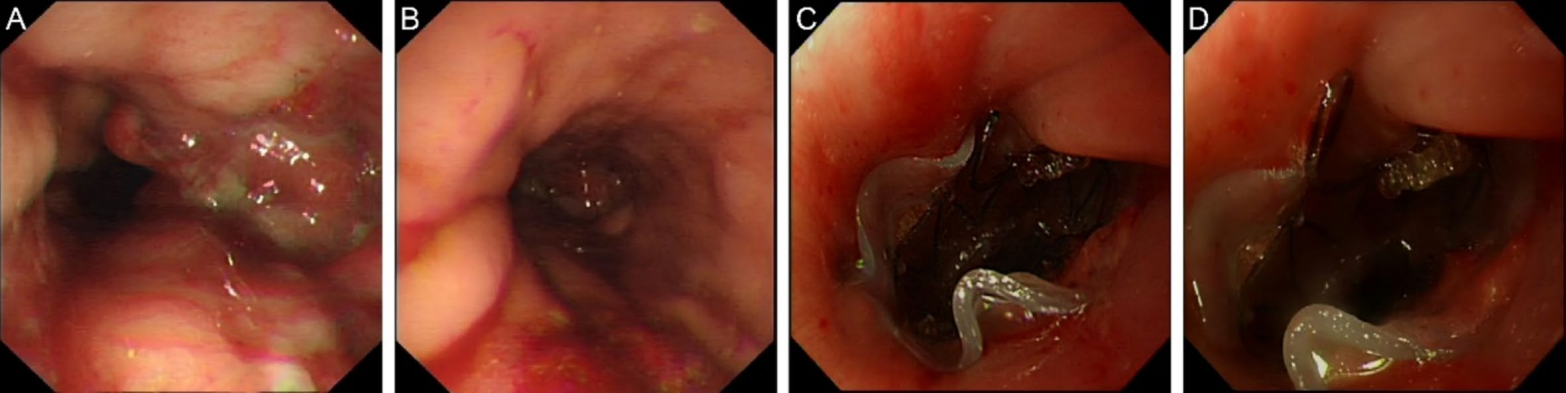
Endoscopic images in May 2024. **(A)** Luminal stenosis image **(B)** Stent removal endoscopic image. **(C)** Stent placement image. **(D)** Follow-up endoscopic image.

Endoscopic exploration on May 7, 2024 showed luminal stenosis approximately 20 cm from the incisors, with the proximal end of the stent at approximately 20.5 cm and the distal end at approximately 29.5 cm from the incisors. The distal end of the stent showed hyperplastic-like elevation and mucosal hyperemia, suggesting stent migration. Anastomotic fistula was detected. After oral administration of meglumine diatrizoate, the mucosa at the anastomotic site appeared slightly disordered, without significant stenosis. A small amount of contrast agent was seen extravasating into the trachea and thoracic stomach, suggesting an esophageal-tracheal fistula. The decision was made to replace the stent, and the stent was removed using a foreign body retrieval hook (Figures 3A,B).

### 2.3 History of past illness

**Figure 4:**
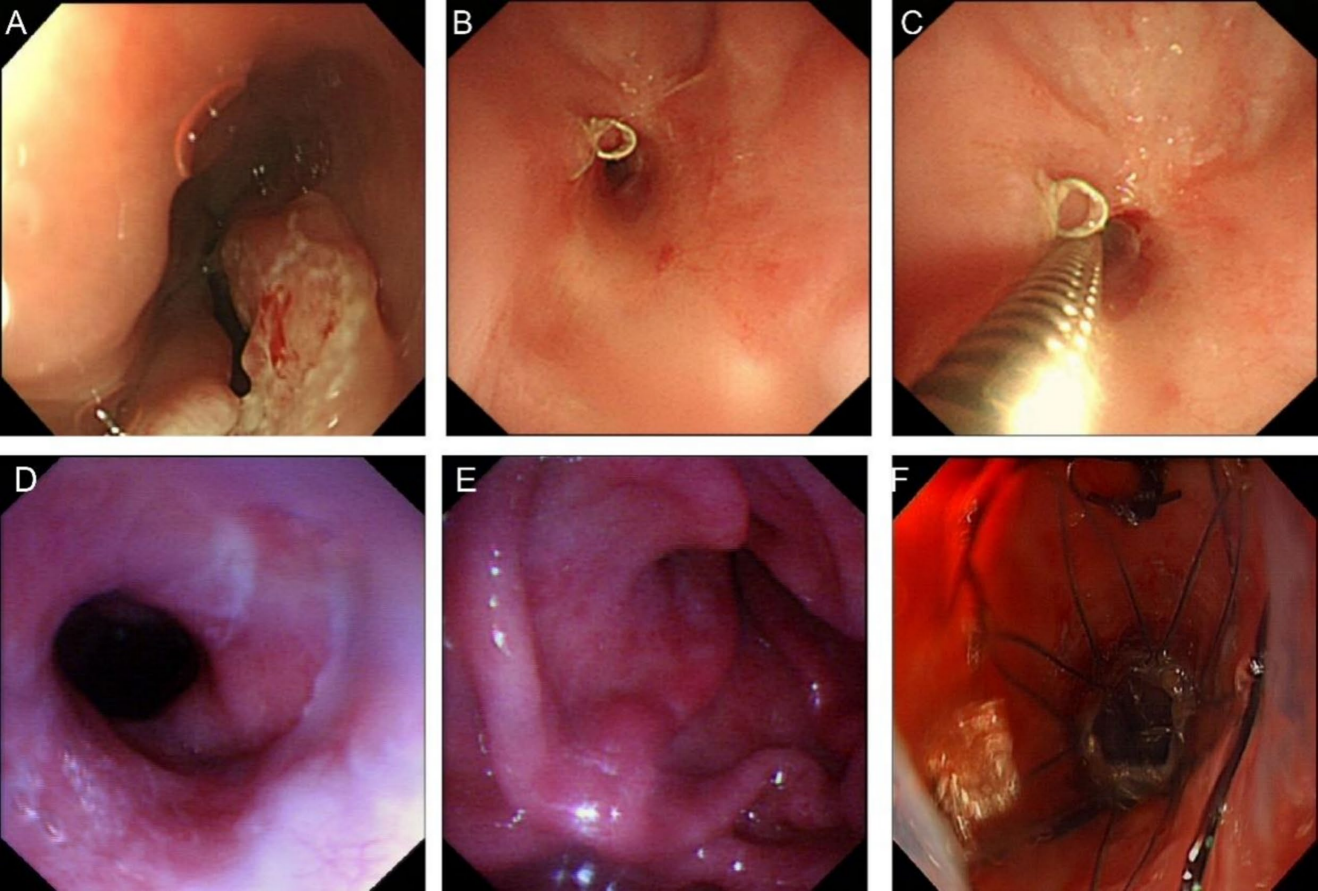
Images of esophageal cancer and stenosis. **(A)** Endoscopic image of esophageal cancer in 2021. **(B)** Stenosis image in 2022. **(C)** Ultra-thin endoscope unpassable image in 2022. **(D)** Esophageal entrance image in October 2022. **(E)** Anastomotic stenosis image in October 2022. **(F)** Stent placement image in October 2022.

A 72-year-old female patient presented with dysphagia on July 21, 2021, and underwent electronic gastroscopy at Jinling Clinical Medical College, Nanjing University of Chinese Medicine (hereinafter referred to as “our hospital”). The gastroscopy revealed irregular elevation of the mucosa on the posterior wall of the esophagus at 28–31 cm, and the diagnosis was esophageal cancer (Figure 4A). Pathological examination of the biopsy taken from the “esophagus at 28–31 cm” confirmed moderately differentiated squamous cell carcinoma. On September 13, 2021, the patient underwent “total thoracoscopic esophageal subtotal resection with cervical esophagogastrostomy” at our hospital. Post-operative pathology confirmed moderately differentiated squamous cell carcinoma (pT2NoMo), and the patient developed a suspected esophageal anastomotic fistula, which was managed with incision opening, double-tube irrigation, and drainage. Follow-up on December 21 showed satisfactory healing, and adjuvant radiotherapy was then initiated.

On February 28, 2022, the patient was admitted to our hospital due to dysphagia that had persisted for 3 days. This dysphagia was caused by postoperative esophageal stenosis resulting from the “total thoracoscopic esophageal subtotal resection with cervical esophagogastrostomy” performed on September 13, 2021, and as a result, the patient was unable to consume food or water. Considering postoperative esophageal stenosis, endoscopic exploration revealed stenosis approximately 20 cm from the incisors, with a diameter of approximately 0.4 cm, making passage with an ultra-thin endoscope impossible (Figures 4B,C). Metal staples were visible above the anastomotic site. Esophageal dilation was performed using 7 mm, 9 mm, 11 mm, and 12 mm dilators sequentially. After dilation, endoscopy showed a patent anastomotic site, and the patient was discharged without discomfort. On several occasions after the initial dilation, the patient experienced recurrent dysphagia. Eventually, on October 17, due to progressive obstruction, the patient requested a stent trial. Endoscopic exploration showed that the esophageal inlet was approximately 17 cm from the incisors, and anastomotic stenosis was located approximately 20–22 cm from the incisors. Esophageal dilation and esophageal stent placement were performed. After dilation with a 1.0 cm dilator, a 15 × 45 mm Sigma esophageal covered metallic stent (anti-reflux, with suture) was placed, with the proximal end of the stent approximately 19 cm from the incisors (Figures 4D–F). The patient had no significant pharyngeal foreign body sensation postoperatively. A follow-up upper gastrointestinal radiography on the second postoperative day showed the stent in place, and the contrast agent entered the residual stomach smoothly. The patient was discharged after successfully initiating oral intake.

On May 22, 2023, esophageal stent placement was performed with an 18 mm × 90 mm Sigma fully covered esophageal metallic stent (Figure 2D), with the proximal end of the stent approximately 18.5 cm from the incisors. The patient had no discomfort after consuming liquids and was discharged.

### 2.4 Personal and family history

The patient denied any family history of malignant tumors.

### 2.5 Physical examination

The patient had stable vital signs, clear consciousness, but poor vitality. The abdomen was flat with postoperative changes in the upper to middle abdomen, and no other significant abnormalities were observed.

### 2.6 Laboratory examinations

The serum tumor markers detected in this examination mainly included carcinoembryonic antigen (CEA) 4.72 μg/L (normal reference value <5 μg/L), squamous cell carcinoma antigen (SCC) 0.45 μg/L (normal reference value <1.5 μg/L), cytokeratin 19 fragment (CYFRA21-1) 1.2 μg/L (normal reference value <3.3 μg/L), etc. The levels of these markers were all within the normal ranges. No abnormality was found in routine blood and urine analyses.

## 3 Treatment

On May 15, 2024, esophageal dilation and stent placement were performed using 9 mm and 11 mm dilators. A Sigma fully covered esophageal metallic stent (Figure 3C) with a length of 8.5 cm, an upper segment diameter of 19 mm, and the remaining diameter of 15 mm was placed, with the proximal end of the stent approximately 18.5 cm from the incisors. Fluoroscopy showed good stent expansion.

## 4 Outcome and follow-up

The patient was discharged without discomfort after final stent placement on May 15, 2024.

### 4.1 Clinical evaluation results

Follow-up gastroscopy in October 2024 showed that the stent position was correct and there were no signs of stenosis or fistula recurrence (Figure 3D). The surrounding mucosa was normal with no signs of inflammation or tissue proliferation. In addition to the gastroscopy, chest X-ray was performed, and it was found that the position of the stent was stable, without signs of displacement and deformation.

### 4.2 Patient evaluation results

Patients reported significant improvement in symptoms. The patient can now eat normally, including solid foods, without choking or vomiting. The frequency of coughing is also greatly reduced, from multiple times a day to occasional episodes, greatly improving patients’ quality of life.

### 4.3 Important follow-up diagnosis and other findings

During follow-up, serum tumor markers, including carcinoembryonic antigen (CEA) and squamous cell carcinoma antigen (SCC), were regularly monitored. The levels of these markers remained within normal ranges, indicating no evidence of tumor recurrence. Routine analysis of blood and urine showed no abnormalities, indicating that the patient’s overall physical condition was stable.

### 4.4 Intervention compliance and tolerability

Patients showed good adherence to the treatment regimen. The patient attended all scheduled follow-up visits on time and strictly followed the dietary and lifestyle recommendations provided by the medical team. Stent placement and esophageal dilation were well tolerated. After each procedure, patients experience only mild discomfort, which resolves on its own within a few days without special treatment.

### 4.5 Adverse and unexpected events

The major adverse event throughout the course of treatment was stent displacement. The patient’s dysphagia symptoms recurred and the cough worsened. Timely action was taken and the stent was successfully replaced. After the replacement, the patient’s symptoms improved rapidly and no further adverse events were observed during subsequent follow-up.

In summary, the patient was treated with a Sigma fully covered metal stent for ultra-high malignant esophagotracheal fistula, and good results were achieved both from clinical symptoms and objective examination results. However, ongoing follow-up is still necessary to ensure long-term stability and to monitor for any potential recurrence or complications.

## 5 Discussion

Esophageal fistula is one of the most life-threatening complications following esophageal cancer surgery. Its main clinical manifestations include recurrent choking, difficulty in swallowing and eating, and a predisposition to uncontrollable persistent respiratory infections. It is difficult to close spontaneously, significantly reducing the patient’s quality of life (6). Due to the unique anatomical location of super-high esophageal-tracheal fistulas, treatment poses a significant challenge. Traditional surgical methods often involve extensive trauma, high risks, and slow postoperative recovery, posing a difficult problem that urgently needs to be addressed in clinical practice (7). Esophageal stent placement, as a palliative treatment, has significant effects in improving patients’ symptoms and quality of life (8). However, traditional esophageal covered stents are prone to migration, cause a strong foreign body sensation, and may lead to tissue hyperplasia with long-term placement, making them difficult to remove and increasing the difficulty of applying this technology. In this case, the Sigma esophageal fully covered stent successfully addressed the challenges of high-level obstruction, multiple fistulas, and stent replacement after long-term placement.

The Sigma stent successfully addresses the issues of stent migration and slippage. The incidence of stent migration and slippage is approximately 2.9–4.3% (9), especially in the treatment of super-high esophageal fistula stenosis (10). In patients with high anastomosis after gastric or esophageal cancer surgery, the dilated residual stomach below the anastomosis lacks support, easily causing stent slippage and leading to treatment failure. In this case, the Sigma stent uses a combination of three methods to address stent migration: ① the upper end of the Sigma stent is designed in a flare shape, with a larger diameter at the upper segment than the lower segment, allowing it to be stably wedged at the anastomotic stenosis. ② Anti-slip barbs. ③ A suspension wire design is used. When the stent is first placed, it is not fully expanded. The suspension wire design prevents it from slipping. After 2 weeks, the wire is cut and removed.

The Sigma stent addresses the issue of a foreign body sensation with high-level stents. Chest pain and a strong foreign body sensation are common complications of super-high metal stents (11). Conventional stents have exposed metal wires, are relatively rigid, and have poor conformability. Especially when placed at a super-high level, the foreign body sensation is often very pronounced, causing patients to be intolerant and leading to surgical failure. The Sigma stent used in this case employs ① a silicone membrane full-coverage design to reduce mucosal irritation. ② The stent is designed in segments, with the oral-side segment controlling the length to 1.5 cm, reducing the wire diameter, decreasing support force, and alleviating the foreign body sensation, making the patient more comfortable.

The Sigma stent does not cause significant tissue hyperplasia. Tissue hyperplasia and stenosis are the primary causes of recurrent dysphagia after stent placement (12). During esophageal motility, the stent edges repeatedly rub against and irritate the esophageal epithelium, causing local esophageal damage, and inflammatory reactions, and inducing the formation of granulation tissue (13). Traditional stents have exposed metal wires, which are prone to tissue adhesion and scarring, making it difficult to remove the stent. In contrast, the Sigma stent used in this case employs a silicone membrane design, which reduces tissue adhesion and allows for removal even after 1 year of placement. Based on our center’s cumulative practical experience, even stents that have been implanted for up to 2 years can still be removed smoothly and safely.

**Figure 5:**
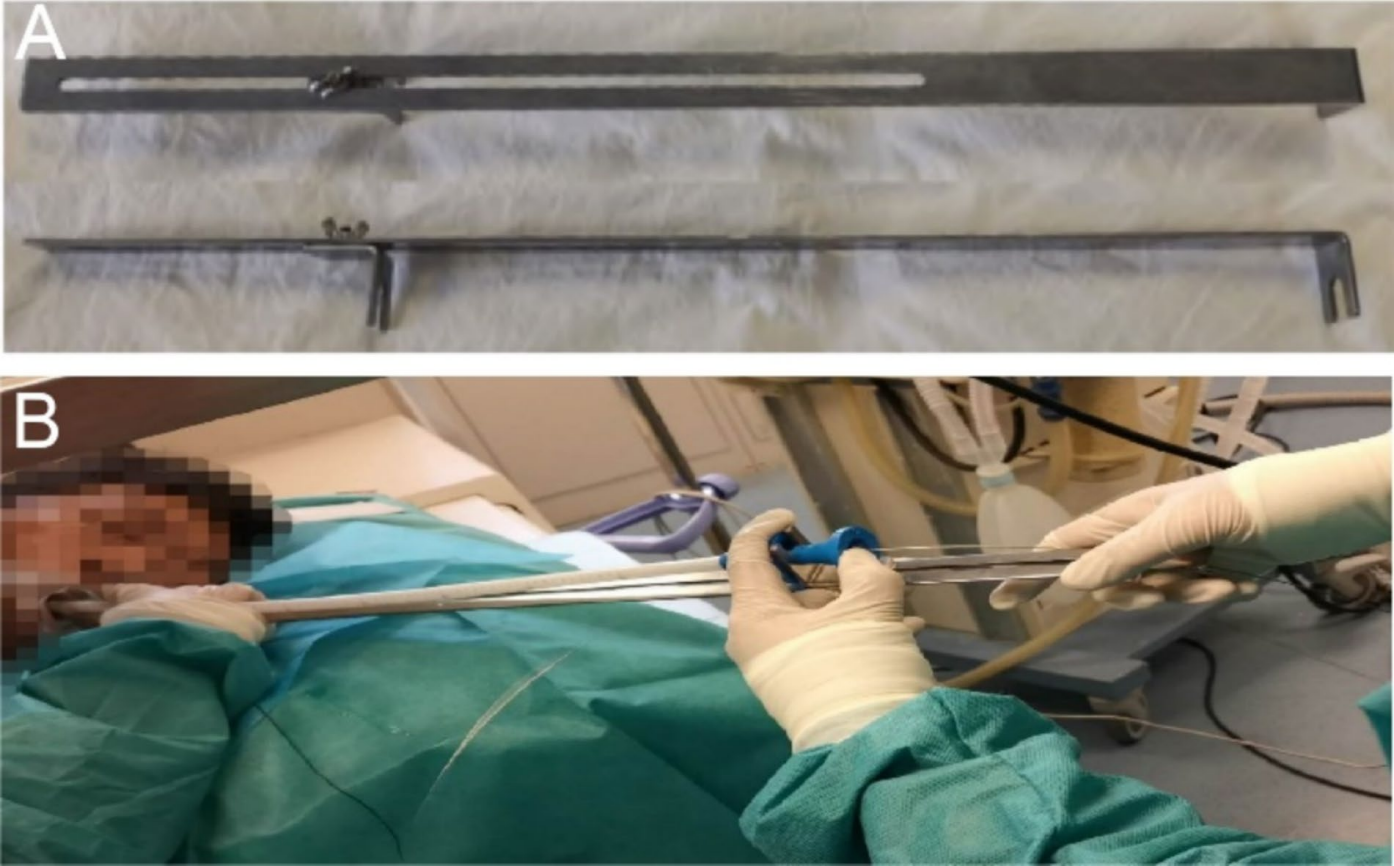
External localization device. **(A)** Localization device image. **(B)** Precise placement image.

The Sigma stent can achieve precise stent deployment using an external localization ruler (Figures 5A,B). In contrast, the placement of traditional stents often relies on X-ray guidance combined with endoscopic manipulation, especially when placing super-high stents, where precision is crucial. Given this, our center uses an external localization ruler tool that enables millimeter-level precise positioning of stent placement in an environment without X-ray radiation, greatly improving the success rate and safety of the surgery. In this case, the upper end of the stent needed to be precisely positioned at a height of approximately 18.5 centimeters from the incisors. The use of the external localization ruler made this technology more convenient and safe.

In the field of esophageal disease treatment, esophageal stent placement has emerged as a significant interventional means (4). In this study, the Sigma fully covered esophageal stent was successfully utilized in the treatment of a super-high malignant esophagotracheal fistula. However, not all studies align perfectly with the outcomes of this case. A similar supporting case report by Lin et al. (5) applied a Y-shaped Sigma stent loaded with I^125^ seeds to patients with inoperable malignant main airway obstruction, resulting in good postoperative airway lumen restoration and significant improvement in patients’ Karnofsky Performance Status (KPS) scores and partial pressure of oxygen in arterial blood (PaO₂). This demonstrates the remarkable efficacy of Sigma stents in addressing airway obstruction issues. Although the application scenarios differ, it provides theoretical support and practical reference for the use of Sigma stents in esophageal disease treatment, suggesting their effectiveness in similar complex luminal diseases.

Conversely, there are also research viewpoints that contradict this case. For instance, the “European Society of Gastrointestinal Endoscopy (ESGE) Guideline on Esophageal Stenting for Benign and Malignant Disease (2021)” (8) states that self-expandable metal stents (SEMSs) are not recommended as first-line treatment for benign esophageal strictures, primarily due to potential adverse event risks, the availability of alternative therapies, and higher costs. While this case primarily focuses on malignant esophagotracheal fistulas and related strictures, it highlights the need for careful consideration when applying different types of stents in esophageal disease treatment. Although Sigma stents performed exceptionally well in this case, they may face different considerations in other scenarios, such as further evaluation of their long-term effectiveness and safety in the treatment of benign diseases. Didden et al. (14) mentions that although endoscopic stent placement rapidly alleviates symptoms in cases of malignant dysphagia and fistulas, recurrent dysphagia and other stent-related complications are common. While Sigma stents successfully addressed challenges such as high obstruction, multiple fistulas, and stent replacement in this case, it underscores that stent treatment is not always straightforward in the broader context of esophageal disease treatment, and even novel stents may encounter various issues, contrasting with the favorable treatment outcomes in this case. Such discrepancies may stem from individual patient differences, such as underlying diseases, physical condition, severity and location of lesions, as well as factors related to treatment procedure details, stent placement timing, and postoperative management.

In summary, existing research provides insights into stent treatment for esophageal diseases from various perspectives. While Sigma stents have achieved success in the treatment of super-high malignant esophagotracheal fistulas in this case, it is essential to recognize the diversity of research outcomes, providing a reference for further research and clinical practice in the future.

## 6 Conclusion

The Sigma esophageal fully covered metal stent is safe and feasible for the treatment of super-high esophageal-tracheal fistulas, with a low incidence of complications. This stent can address recurrent stenosis and diseases at high anastomotic sites, can be removed even after long-term placement, and can significantly improve patients’ symptoms and quality of life. Therefore, for patients with super-high esophageal-tracheal fistulas following esophageal cancer surgery or radiotherapy, Sigma esophageal metal stent placement may be a good option for endoscopic treatment in this high-risk patient population.

## 7 Prospect

Future efforts will further enhance stent stability, increase patient education post-stent placement (e.g., small, slow swallowing), and follow-up with regular stent replacements and adjustments in diameter. Additionally, to further validate the effectiveness and safety of the stent, we plan to increase the sample size for research. In summary, we are committed to continuously improving the efficacy and safety of stent treatment through technological innovation and clinical practice, bringing better treatment experiences and outcomes to patients.

## 8 Definition

Super-high esophageal fistulas refer to esophageal fistulas that occur in the upper esophagus and are 15 to 23 cm from the incisor under endoscopy.

## Core tip

Esophageal cancer treatment often leads to stenosis and fistulas. This case report presents a 72-year-old female patient with super-high esophageal-tracheal fistula post-surgery, successfully treated with Sigma fully covered metallic stent placement. Despite stent migration and recurrent stenosis, repeated stent placements resolved symptoms, highlighting the efficacy and challenges of this clinical approach.

## Data Availability

The original contributions presented in the study are included in the article/supplementary material, further inquiries can be directed to the corresponding authors.
